# Use of DORA Daridorexant for the deprescription of hypnotic medications for chronic insomnia comorbid with mood,anxiety and sedative hypnotic use disorders: a naturalistic longitudinal study

**DOI:** 10.1192/j.eurpsy.2025.505

**Published:** 2025-08-26

**Authors:** L. Anastasio, G. Alfi, G. Aquino, E. Annuzzi, G. Cerofolini, A. Coccoglioniti, M. Gambini, M. Pioltino, G. Perugi, A. Gemignani, L. Palagini

**Affiliations:** 1 Department of Neuroscience, Psychiatric Unit, Azienda Ospedaliera Universitaria Pisana (AUOP), University ofPisa, Pisa, Italy; 2 University of Pisa, Pisa, Italy

## Abstract

**Introduction:**

Targeting insomnia may have a therapeutic value for mental disturbances. Indeed, insomnia chronic nature may necessitate switching between various insomnia approaches and medications. Daridorexant is a new *pharmacological option for insomnia treatment,* which is a *dual orexin receptor antagonist (DORA).*

**Objectives:**

*The* aim of the present study was to treat consecutive patients with insomnia disorder with and without mental co-morbidities with this new therapeutic option and during the deprescripion of previous hypnotic medications which have failed in insomnia treatment.

**Methods:**

Ninety consecutive patients with insomnia disorder according to the DSM-5-TR criteria (sleep condition indicator SCI<16) were treated with daridorexant 50 mg. Baseline, 1 month, and 3 month evaluations were performed. Demographic and clinical data were incorporated. Insomnia severity (Insomnia Severity Index-ISI), mood and anxiety symptoms (Beck Depression Inventory II-BDI-II, Young Mania Rating Scale-YMRS, Self Reported Anxiety Scale-SAS), and pre sleep arousal(Pre Sleep arousal Scale-PSAS) were evaluated. The evaluation of psychiatric diagnosis was conducted in accordance with the DSM-5-TR criteria (SCID-5) and the concurrent use of pharmacological therapy was taken into account. For previous insomnia treatment discontinuation, cross-tapered programs were developed following the 25% per week rule while inserting daridorexant.

**Results:**

The final sample included 80 patients (N° 40, 50.0% females, mean age 60 ± 13.2). Most of them (75.5%) suffered from insomnia comorbid with unipolar/bipolar depression, anxiety disorders and sedative-hypnotics use disorders. Repeated Anova analyses showed that ISI and PSAS total score decreased across time (respectively F=63.42, p<0.001, F=61.44, p<0.001). Similarly mood symptoms, anxiety symptoms significantly improved over time and after 3 months of treatment (respectively F=62.45, p<0.001, F=31.48, p<0.001, F=41.14, p<0.001, F=21.44, p<0.001). Analyses conducted on a subsample of patients with comorbid sedative-hypnotics use disorders (50%) revealed a beneficial effect on insomnia, arousal and anxiety symptoms. Multiple regression models demonstrated that the clinical improvement of anxiety symptoms was most best predicted by the improvement in emotion regulation -PSAS score at T1 and T2, and the improvement in insomnia symptoms at T1and T2.

**Conclusions:**

The treatment of chronic insomnia with daridorexant improved insomnia symptoms in patients with and without mental comorbidities across time. It was particularly effective in patients with sedative-hypnotics use disorders, where we deprescribed previous sedative hypnotics including neuroleptics and sedative antodepressants. These data are in line with the data showing that sleep is involved in the regulation of the reward system.

**Disclosure of Interest:**

L. Anastasio: None Declared, G. Alfi: None Declared, G. Aquino: None Declared, E. Annuzzi: None Declared, G. Cerofolini: None Declared, A. Coccoglioniti: None Declared, M. Gambini: None Declared, M. Pioltino: None Declared, G. Perugi: None Declared, A. Gemignani: None Declared, L. Palagini Consultant of: Consultancy for Laura Palagini, Bruno, Idorsia, Fidia, Pfizer, Sanofi

